# Genomic, transcriptomic and metabolomic analyses of *Amorphophallus albus* provides insights into the evolution and resistance to southern blight pathogen

**DOI:** 10.3389/fpls.2024.1518058

**Published:** 2025-02-07

**Authors:** Longfei Duan, Jianfeng Qin, Gaoxin Zhou, Chuan Shen, Baofu Qin

**Affiliations:** ^1^ Laboratory of Life Sciences, College of Life Sciences, Northwest A&F University, Xianyang, Shaanxi, China; ^2^ Ankang Academy of Agricultural Sciences, Ankang, Shaanxi, China; ^3^ College of Economics and Management, Ankang University, Ankang, Shaanxi, China

**Keywords:** *Amorphophallus albus*, genome evolution, transcriptomic and metabolomic, whole genome duplication, *Sclerotium rolfsii* (SR)

## Abstract

**Introduction:**

*Amorphophallus albus*, a perennial herb in the *Araceae* family, is a valuable cash crop known for its high production of konjac glucomannan and high disease resistance.

**Methods:**

In this study, we present a high-quality, chromosome-scale genome assembly of *A. albus* using a combination of PacBio HiFi sequencing, DNBSEQ short-read sequencing, and Hi-C technology. To elucidate the molecular mechanisms underlying southern blight resistance, we performed an integrated analysis of transcriptomic and metabolomic profiles across three infection stages of *A. albus*.

**Results and discussion:**

Here, we assembled and annotated the complete genome of *A. albus*, providing a chromosome-level assembly with a total genome size of 5.94 Gb and a contig N50 of 5.61 Mb. The *A. albus* genome comprised 19,908 gene families, including 467 unique families.The slightly larger genome size of *A. albus* compared to *A. konjac* may have been affected by a recent whole-genome duplication event. Transcriptional and metabolic analyses revealed significant enrichment of differentially expressed genes (DEGs) and differentially accumulated metabolites (DAMs) involved in phenylpropanoid biosynthesis, plant hormone signal transduction, phenylalanine metabolism, and the biosynthesis of phenylalanine, tyrosine, and tryptophan. These findings not only advance the understanding of genetic and evolutionary characteristics of A. albus but also provide a foundation for future research on the resistance mechanisms of *konjac* against southern blight disease.

## 1 Introduction

Konjac (*Amorphophallus* spp.), a perennial herbaceous plant belonging to the Araceae family, comprises over 220 species that have been cultivated for centuries in East and Southeast Asia, particularly in China and Japan (Ulrich et al., 2016; Shen et al., 2024). The corm of konjac is rich in glucomannan (KGM), a soluble dietary fiber with numerous health benefits and industrial applications (Behera and Ray, 2016). In recent years, konjac has garnered increasing attention due to its potential in food, pharmaceutical, and cosmetic industries, as well as its role in sustainable agriculture and ecological restoration (Chua et al., 2010; Fang et al., 2023). Despite its economic and ecological importance, genomic resources for konjac remain limited, hindering our understanding of its evolutionary history, genetic diversity, and the molecular mechanisms underlying important agronomic traits. Recently, a study reported the genome of *Amorphophallus konjac*, one of the most widely cultivated konjac species worldwide (Gao et al., 2022). High-quality genome assemblies have become essential tools for advancing crop improvement, facilitating comparative genomics, and elucidating the genetic basis of adaptive traits (Li et al., 2023). For instance, it enables the development of molecular markers for marker-assisted selection, the identification of quantitative trait loci (QTLs) associated with desirable traits, and the potential for genomic selection in breeding programs (Zhang et al., 2022).

Recent advancements in sequencing technologies and bioinformatics algorithms have enabled the generation of chromosome-scale genome assemblies for various plant species, providing unprecedented insights into plant evolution, domestication, and trait improvement (Kong et al., 2023). The integration of long-read sequencing technologies, such as Pacific Biosciences (PacBio) and Oxford Nanopore, with short-read Illumina sequencing and chromosome conformation capture (Hi-C) techniques has emerged as a powerful approach for constructing high-quality, chromosome-level genome assemblies (Jiao and Schneeberger, 2017; Li et al., 2019). This multi-platform strategy effectively addresses the challenges posed by repetitive sequences, structural variations, and heterozygosity in plant genomes, resulting in more complete and accurate assemblies compared to traditional short-read-based approaches (Rabanus-Wallace et al., 2021; Yao et al., 2022; Zhang et al., 2023).

Genome assemblies serve as foundational resources for various genomic analyses, including the identification of whole-genome duplication (WGD) events, gene family expansions and contractions, and species-specific adaptations (Feng et al., 2022; Li et al., 2022). For instance, analysis of synonymous substitution rates (Ks) distributions can reveal ancient polyploidization events and estimate divergence times between species (Song et al., 2020). Moreover, comparative genomic analyses enable the identification of conserved and lineage-specific genes, providing insights into the evolution of important traits and metabolic pathways (Gao et al., 2018; Qiao et al., 2019).

*A. albus* is another widely cultivated konjac species, characterized by relatively smaller plants and lower yields compared to *A. konjac* (Gao et al., 2021). However, *A. albus* corms contain the highest KGM content among all konjac species, making it a crucial pillar industry for poverty alleviation in the Shaanxi, Sichuan, and Yunnan provinces of China (Yue et al., 2021; Niu et al., 2024). Notably, *A. albus* exhibits resistance to southern blight disease, a severe pathogen that can significantly decrease the quality and yield of konjac crops (Gao et al., 2023). Despite its agronomic importance, the resistance mechanism of *A. albus* remains unknown, primarily due to the lack of a reference genome.

A high-quality genome assembly of *A. albus* is crucial for elucidating the genetic basis of disease resistance mechanisms and secondary metabolite pathways. Such genomic resources could facilitate targeted breeding efforts, potentially leading to the discovery of novel bioactive compounds with pharmaceutical or industrial applications (Mochida et al., 2017; Varshney et al., 2021). Moreover, a comprehensive genomic analysis could provide insights into the evolutionary history of the Araceae family, particularly considering the unique morphological and physiological adaptations found in konjac species (Cusimano et al., 2011).

In this study, we present a high-quality, chromosome-scale genome assembly of *A. albus* using a combination of PacBio HiFi sequencing, DNBSEQ short-read sequencing, and Hi-C technology. Our comprehensive analysis encompasses genome annotation, comparative genomics, and an in-depth investigation of key gene families associated with disease resistance. To elucidate the molecular mechanisms underlying southern blight resistance, we performed an integrated analysis of transcriptomic and metabolomic profiles across three infection stages of *A. albus*, with particular emphasis on resistance-related genes and metabolites. This integrative approach revealed significant insights into gene expression patterns, evolutionary processes, and gene family expansion/contraction dynamics. Our findings not only advance the understanding of *A. albus* biology but also provide valuable genomic resources for future crop improvement programs.

## 2 Materials and methods

### 2.1 Genome sequencing and assembly

**Figure 1:**
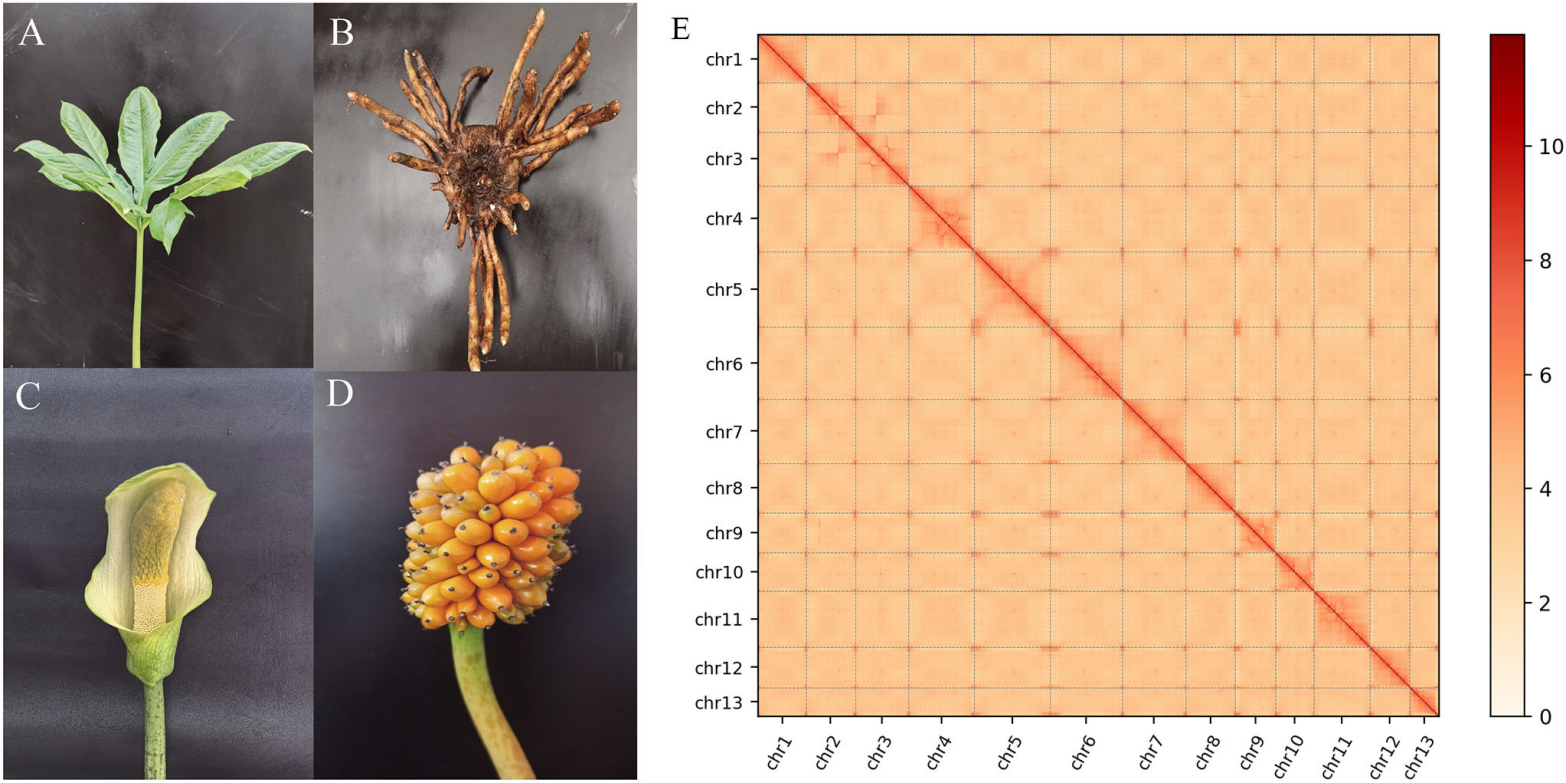
Overview of the *A.albus* genome. **(A-D)** represents respectively the leaf, corm, flower and fruit morphyology of *A. albus*; **(E)** Hi-C heat map of chromosome interactions. The x and y axes represent the ordered positions of the chromosomes of the genome.

The leaves of 1 years old *A. albus* (AnBaiYu-1) (**Figure 1A**) were selected as the materials for genome analysis. The *A. albus* named Anbaiyu-1 (**Figures 1A–D**) was independently bred by the Ankang Academy of Agricultural Sciences. The plant was cultivated in the greenhouse of Ankang Academy of Agricultural Sciences Shaanxi province, China.

High-quality genomic DNA was extracted from *A. albus* leaves using an optimized cetyl-trimethylammonium bromide (CTAB) method. DNA quantity and purity were subsequently evaluated with a Qubit Fluorometer and Nano Drop spectrophotometer Genomic sequencing libraries of *A. albus* was performed using the DBSSEQ and PacBio HiFi platforms. For DBSSEQ sequencing, a short-read library (300-400 bp) was prepared and sequenced on the DBSSEQ platform to obtain high-quality clean reads. In parallel, a long-read library was constructed from genomic DNA and sequenced on the PacBio platform, with low-quality reads and adapter sequences removed to yield clean subreads. Genome size estimation for *A. albus* was conducted through K-mer distribution analysis based on DBSSEQ data. K-mer frequencies were rapidly counted using Jellyfish (v2.2.6), and then genome size, heterozygosity, repeat sequences, sequencing depth, and other genome characteristics were estimated using GenomeScope v2.01 based on the K-mer frequency results.

To assess the assembly quality of the *A. albus* genome, Hi-C technology was employed to facilitate contig anchoring. Assembly completeness was evaluated using Benchmarking Universal Single-Copy Orthologs (BUSCO) v10.

### 2.2 Genome annotation

Genome repeat sequences were identified *de novo* using Repeat Modeler software (Zhao and Hao, 2007). Non-redundant LTR sequences were obtained using LTR FINDER parallel (Tarailo-Graovac and Chen, 2009). Transposable element (TE) protein sequences were predicted with Repeat Protein Mask to identify TE proteins.

Gene structure prediction was conducted through *de novo*, homology-based, and transcriptome-based approaches. *De novo* predictions were generated using Augustus software, applied to genomes masked for repeat sequences. Homology-based predictions utilized protein sequence data from *Spirodela polyrhiza*, *Amorphophallus konjac*, *Selaginella moellendorffii*, and *Physcomitrium patens*. Transcriptome-based predictions were derived from RNA-seq data, mapped to the reference genome using HISAT2 and String Tie. Genome annotation completeness was evaluated with BUSCO5 (Holt and Yandell, 2011). The gene sets from these three prediction methods were integrated using MAKER2 software (Manni et al., 2021). Functional annotation and pathway information for predicted proteins were identified by comparing sequence and motif similarities against Uni Prot, GO, KEGG, Inter Pro, Swiss Prot, Tr EMBL, Pfam, NR, GOG, KOG, and Plant TFDB databases.

Multiple approaches were employed to identify noncoding RNAs in the *A. albus* genome. Transfer RNAs (tRNAs) were predicted using tRNA scan-SE. Small nuclear RNA (snRNA) and ribosomal RNA (rRNA) genes were identified by searching the Rfam database with Infernal software. MicroRNA (miRNA) genes were annotated using BLASTN, referencing the miRBase dataset (Nawrocki and Eddy, 2013).

### 2.3 Phylogenetic tree construction

A phylogenetic tree was constructed using the genomes of 11 species: *Amorphophallus albus, Amorphophallus* konjac, *Eucalyptus grandis*, *Arabidopsis thaliana*, *Oryza sativa*, *Sorghum bicolor*, *Populus trichocarpa*, *Zostera marina*, *Solanum tuberosum*, *Spirodela polyrhiza* and *Physcomitrium patens.* Orthologous genes among *A. albus* and these species were identified with OrthoFinder. A maximum-likelihood (ML) phylogeny was then inferred using RAxML v8.2.12 (Stamatakis, 2014). Species divergence times were estimated with MCMC Tree v4.9, calibrated against time estimates from the Time Tree database (http://www.time-tree.org/).

### 2.4 Gene family contraction and expansion

Gene family expansion and contraction were analyzed using CAFE v4.2 (Han et al., 2013). Based on the species phylogenetic tree and gene family clustering results, a random birth-death model was applied to estimate the number of ancestral gene family members in each branch, predicting the contraction and expansion of gene families relative to ancestral lineages. Significant expansions or contractions were defined by p-values < 0.05, and functional enrichment analysis was conducted on gene families with significant expansion or contraction.

### 2.5 Whole-genome duplication analysis

To investigate whole-genome duplication (WGD) events, the genomes of *A. albus*, *A. konjac*, *E. grandis*, *A. thaliana*, *O. sativa*, *S. bicolor*, *P. trichocarpa*, *Z. marina*, *S. tuberosum*, *S. polyrhiza*, and *P. patens* were analyzed. WGDI v0.5.6 was utilized to generate a distribution of Ks values, with Gaussian mixture models used to fit the Ks distribution curves. To assess collinearity between *A. albus* and *A. konjac*, syntenic blocks were identified using MCScanX. Genome collinearity between *A. albus* and related species was also evaluated with MCScanX (Wang et al., 2012). Homologous genes between species were identified through BLASTP using default parameters.

### 2.6 RNA-seq and data analysis

RNA-seq analyses of *A. albus* following SR infection were conducted at 0, 12h, 24h, and 48h post-inoculation, including control samples inoculated with PDA. Petiole samples were collected from a 1.0 cm distance from the lesion or inoculation site, with six biological replicates per treatment. All samples were immediately frozen in liquid nitrogen and stored at -80°C for later use. Sequencing was performed on the Illumina platform, with clean data obtained following quality filtering. Reads were mapped to the reference genome, and database quality was evaluated. Differentially expressed genes (DEGs) were identified across sample groups based on gene expression, annotated, and enriched. DESeq2 was used to analyze expression differences, applying a threshold of fold change (FC) ≥2 and a false discovery rate <0.1 (Love et al., 2014). DEGs were annotated and enriched using the GO and KEGG databases (Ashburner et al., 2000; Kanehisa and Goto, 2000).

### 2.7 Metabolite extraction and data analysis

Metabolite analyses of *A. albus* following SR infection were conducted at 0, 12h, 24h, and 48h post-inoculation, including control samples inoculated with PDA. Petiole samples were vacuum freeze-dried and analyzed using LC–MS. Raw LC–MS data files were converted to mzXML format using ProteoWizard software, and peaks with a detection rate below 50% in any sample group were discarded. Metabolite identities were assigned by referencing a custom database integrated with public databases. Variable importance in projection (VIP) values were calculated using OPLS-DA in the R package MetaboAnalystR, with score plots and permutation plots generated. Metabolite identification was performed using the KEGG compound database, and annotated metabolites were subsequently mapped to the KEGG Pathway database.

### 2.8 Combined transcriptome and metabolome analysis

Prioritizing genes and metabolic pathways with a P-value <0.05 enables efficient data screening and facilitates rapid identification of pathways relevant to the research focus for further analysis. Genes were grouped based on differential expression, and correlation coefficients between all genes and metabolites were calculated using Pearson correlation. Data were preprocessed with Z-score transformation prior to correlation analysis, and screening was conducted based on correlation coefficient (CC) and corresponding P-values.

## 3 Results

### 3.1 Genome sequencing and assembly

The genome size, repeat size and heterozygosity of A. albus were estimated using k-mer analysis. The 25-mer frequency distribution of DBSSEQ short reads showed the highest peak at a depth of 129.31. The genome size was estimated to be 5.72 Gb with 76.13% repeats. The heterozygosity rate was estimated at 2.08% (**Supplementary Table S1**).

The genome of *A. albus* was sequenced using the Sequel II sequencing platform, which provided high-quality long reads for the assembly process. The sequencing data was processed to generate 3,051 contigs, which were then organized into 13 pseudo-chromosomes using Hi-C technology. Hi-C scaffolding allowed for the accurate anchoring and ordering of contigs into chromosome-scale structures. This method also helped in constructing whole-genome interaction maps, providing insights into the genome’s 3D structure and confirming the interaction patterns consistent with previously established genome interactions (**Figure 1E**). The final assembly of the *A. albus* genome was 5.94 Gb in size, with a high level of continuity indicated by a contig N50 of 5.61 Mb and a scaffold N50 of 467.26 Mb. These values indicate that the majority of the genome is contained within large, high-quality contigs and scaffolds. The GC content of the genome was found to be 44.87%, which is typical for plant genomes. Despite the high-quality assembly, a total of 2,246 gaps were present across the genome, with the number of gaps per chromosome varying from 62 to 321. These gaps often occur in regions of the genome that are difficult to sequence, such as those with high repetitive content or complex secondary structures (**Supplementary Table S2**).

BUSCO analysis found 1,569 (97.21%) complete gene models and 11 (0.68%) fragmented gene models out of a total of 1,614 genes (**Supplementary Table S3**). These results indicate that our genome assembly of *A. albus* is of high quality.

### 3.2 Repeat and genome annotation

Using a combination of *de novo* and homology-based approaches, 63.83% of the assembled sequences were identified as repetitive sequences, including 58.66% LTR retrotransposons and 2.49% genome DNA transposons (**Supplementary Table S4**).

A total of 35,155 genes were predicted using a combination of *de novo*, homology-based, and transcriptome-based approaches with MAKER software. The average gene length was 12,338 bp, and the average CDS length was 1,028 bp, with an average exon length of 348.38 bp (**Table 1**; **Supplementary Table S5**).

**Table 1:** Statistics for the genome assembly of *Amorphophallus albus*.

Genome information	(PacBio + Hi-C)
Sequencing platform	PacBio Sequel
Genome size (Gb)	5.94
Scaffold number	304
GC content (%)	44.87
N50 (contig) (Mb)	5.61
Predicted Chromosome genes	35155

**Figure 2:**
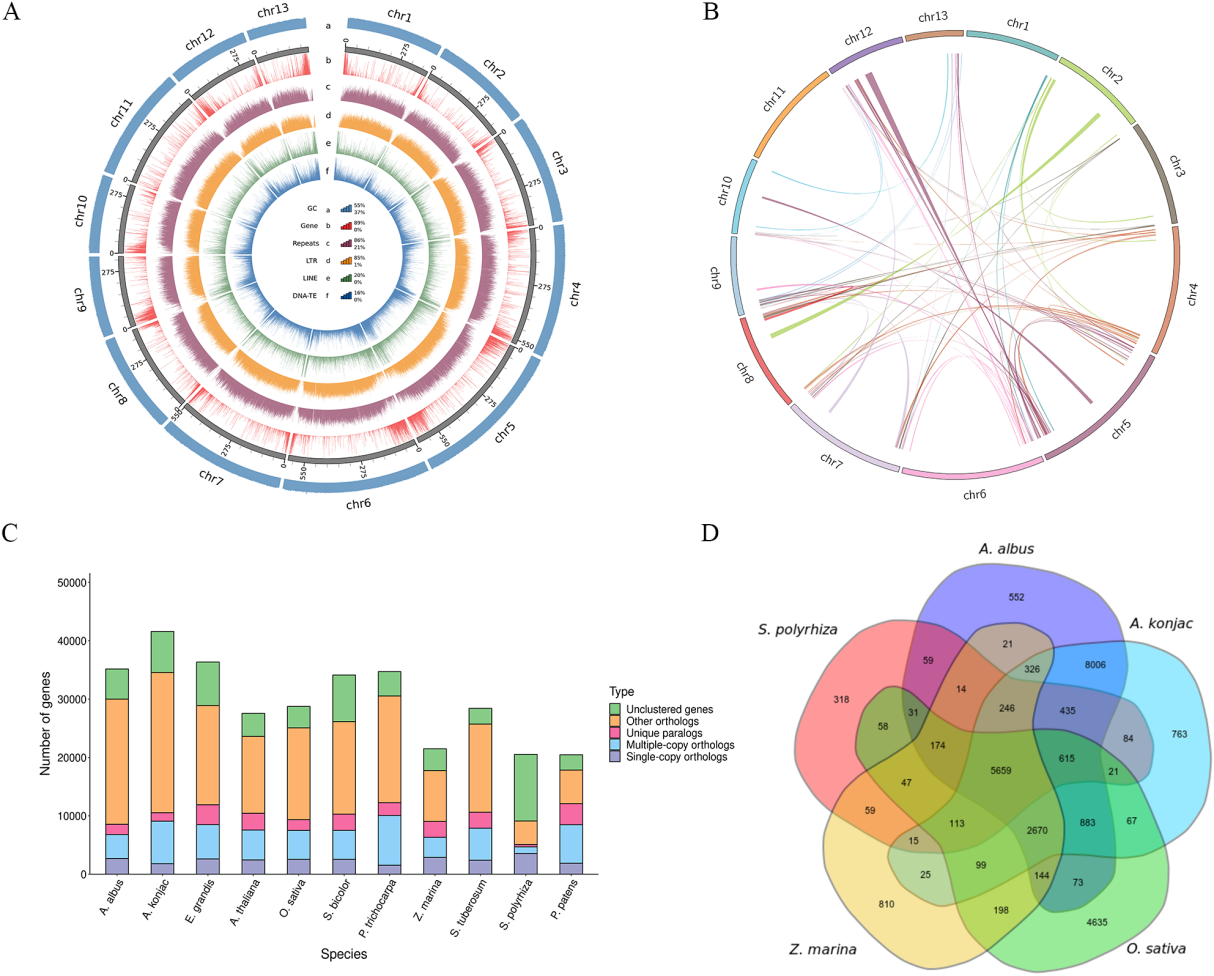
Comparative genomic analysis of the genome of *A. albus*. **(A)** Chromosomal features of *A. albus.* (a) The distribution of GC content across the chromosomes. (b) The density of genes along the chromosomes. (c) The density of repetitive DNA sequences within the genome. (d) The density of LTR retrotransposons across the genome. (e) The density of LINE elements in the genome. (f) The density of DNA transposable elements across the chromosomes. **(B)** Syntenic blocks on homologous chromosomes between *A. albus* and other species. **(C)** The number of homologous genes shared between different species. **(D)** Clustering of common and species-specific gene families in *A. albus*, *A. konjac*, *O. sativa*, *Z. marina*, and *S. polyrhiza*. The clustering shows the evolutionary relationships and unique gene families in each species.

In light of observed GCdensity, gene density, repetitive sequences density, LTR density, LINE density, DNA-TE density and Syntenic block, Two circos map of genome of *A. albus* was drawn (**Figures 2A, B**). Overall, the functions of 31139 genes (88.58%) were assigned. Among these, 28875(89.68%) and 16442(59.27%) had predicted homologs in NR and SwissProt databases, respectively (**Supplementary Table S6**).

### 3.3 Comparative genomic analysis

Genomes from eleven species were selected to identify homologous genes, perform gene family clustering analysis, and examine the enrichment of single-copy genes with *A. albus* (**Figure 2C**). The *A. albus* genome comprised 19,908 gene families, including 467 unique families (**Supplementary Table S7**).

Furthermore, specific and common gene families were analyzed among *A. albus* and four other species. A total of 27,220 genes were clustered among *A. albus*, *A. konjac*, *O. sativa*, *Z. marina*, and *S. polyrhiza*, with 5,659 genes shared across all five species. *A. albus* and *A. konjac* exhibited a close relationship, sharing the highest number of gene families. Additionally, 552 specific gene families were identified in *A. albus*, which is fewer than the 763 specific families identified in *A. konjac* (**Figure 2D**).

### 3.4 Phylogeny of *A.ablus*

**Figure 3:**
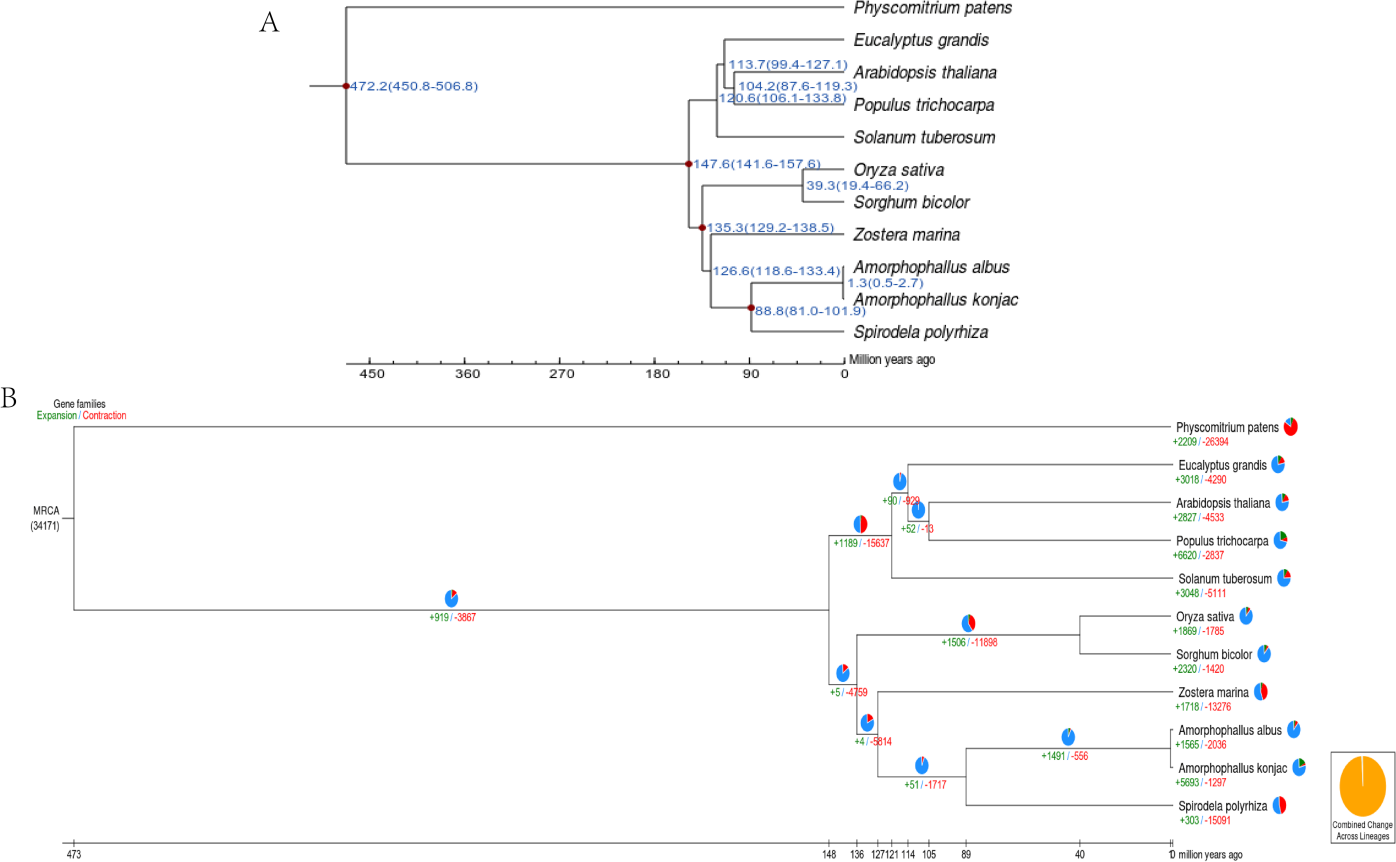
Genome evolution of *A. albus* genome. **(A)** Divergence time estimation. This panel shows the estimated divergence times between *A. albus* and other species. **(B)** Gene family expansion/contraction. The numbers marked in red and green represent the contraction and expansion of gene families, respectively.

To estimate the divergence time among the 11 species, a phylogenetic tree was constructed (**Figure 3A**). The analysis indicated that *A. albus* and *A. konjac* diverged from their common ancestor approximately 1.3 million years ago (mya).

Gene family expansion and contraction play a key role in phenotypic adaption during speciation. There were 2036 gene family contractions and 1565 gene family expansions detected in *A.ablus*, while 297 gene contractions and 5693 gene family expansions were identified in *A.konjac* (**Figure 3B**).

GO gene family enrichment analysis revealed that contracted gene families were primarily associated with ATP hydrolysis activity, heme binding, and monooxygenase activity, while expanded gene families were involved in meristem development, meristem maintenance, and DNA integration (**Supplementary Figures S1**, **S2**). KEGG gene family enrichment analysis indicated that contracted gene families were mainly related to starch and sucrose metabolism, plant-pathogen interactions, and ABC transporters, whereas expanded gene families were associated with the biosynthesis of unsaturated fatty acids, amino sugar metabolism, and phenylpropanoid biosynthesis (**Supplementary Figures S3**, **S4**).

### 3.5 Whole genome duplications of *A.ablus*

**Figure 4:**
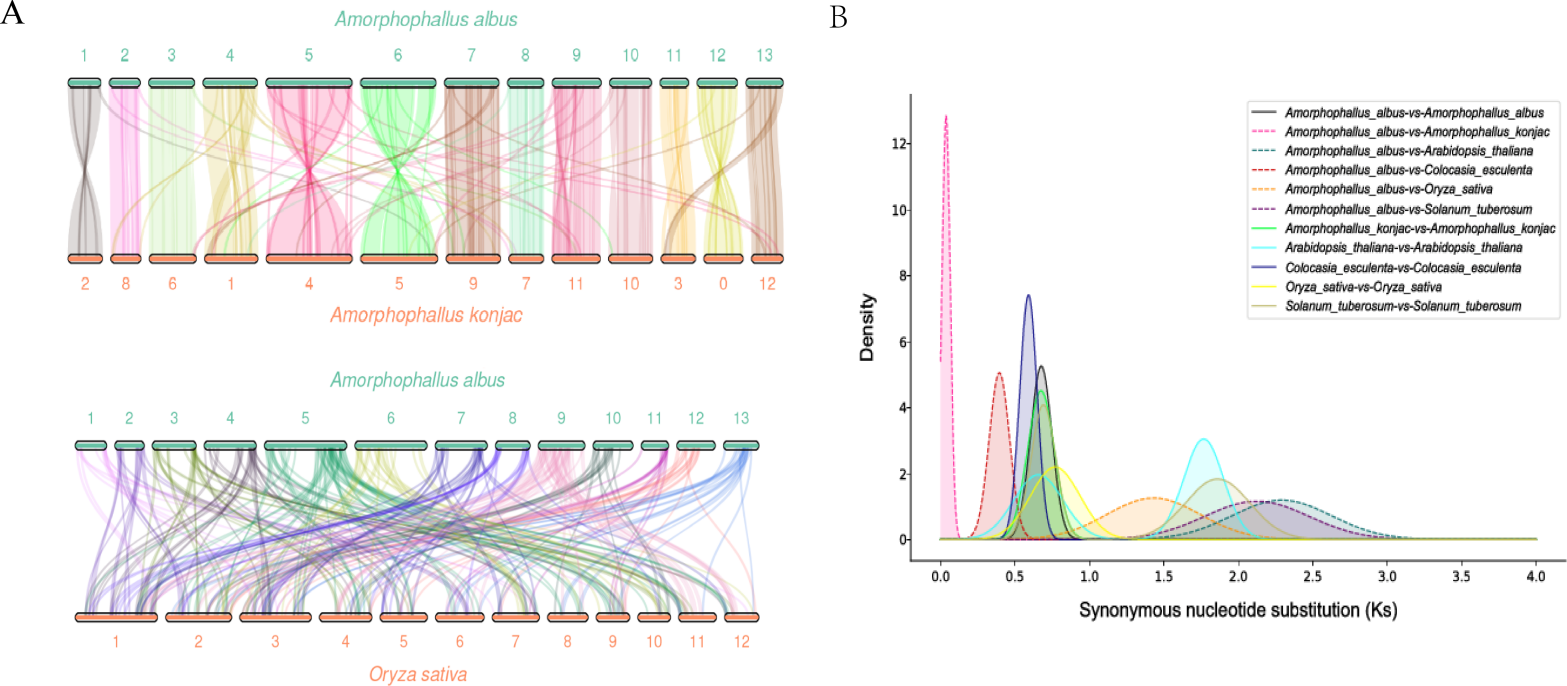
Collinearity diagram and Whole Genome Duplication. **(A)** Collinearity diagram. This diagram illustrates the synteny between *A*. *albus* and other species. **(B)** WGD (Whole Genome Duplication) analysis diagram. This diagram displays the occurrence and distribution of whole genome duplication events within the *A. albus* genome.

The density distribution revealed that *A. konjac* shared the smallest peak with *A. albus*, near 0.69. Based on synonymous nucleotide substitution rates, it was determined that *A. albus* has undergone a whole-genome duplication (WGD) event (**Figure 4B**). Additionally, collinearity analysis of the *A. albus* and *A. konjac* genomes (**Figure 4A**) indicated that both species experienced the same WGD events.

### 3.6 Transcriptome analysis of *A.albus* responsive to SR infection

**Figure 5:**
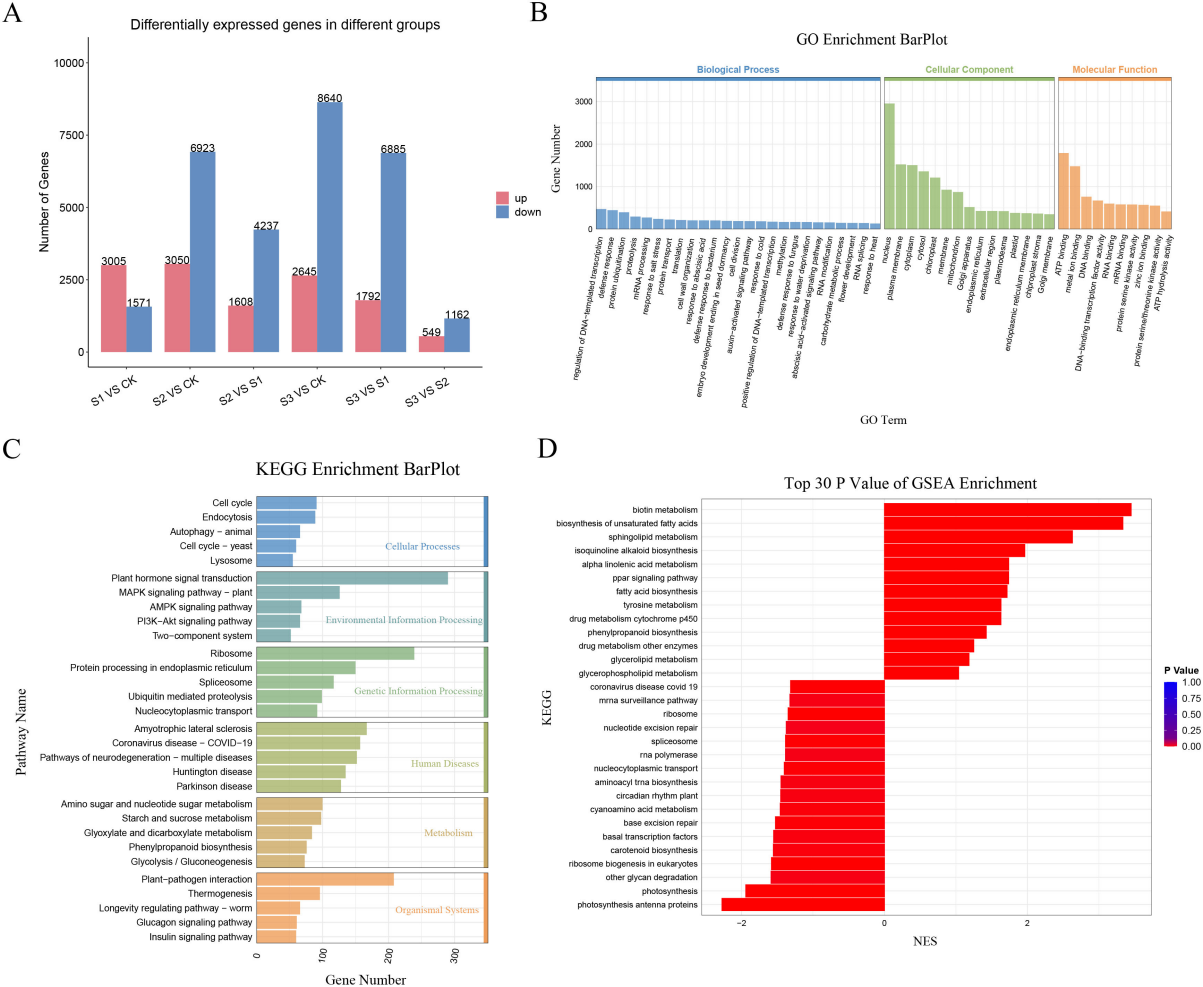
Transcriptome analysis of *A*. *albus* responsive to SR infection during different stages (0h, 12h, 24h, 48h). **(A)** Differentially expressed genes (DEGs) of *A*. *albus* responsive to SR infection. This panel shows the genes that are differentially expressed at various time points (0h, 12h, 24h, and 48h) following SR infection. **(B)** Gene Ontology (GO) enrichment analysis with DEGs of *A. albus* responsive to SR. This panel presents the functional enrichment of DEGs, categorized into different GO terms. **(C)** KEGG enrichment analysis with DEGs of *A*. *albus* responsive to SR. This panel shows the Kyoto Encyclopedia of Genes and Genomes (KEGG) pathway enrichment of the DEGs. **(D)** Gene Set Enrichment Analysis (GSEA) with DEGs of *A. albus* responsive to SR. This panel illustrates the results of GSEA, which identifies the biological pathways and gene sets that are differentially activated in response to SR infection at different time points.

Gene expression profiling of *A. albus* at different SR infection stages (0, 12h, 24h, and 48h) was conducted using RNA-Seq. Six comparative DEG analyses (S1 vs. CK, S2 vs. CK, S2 vs. S1, S3 vs. CK, S3 vs. S1, and S3 vs. S2) identified 4,576, 9,973, 5,845, 10,485, 8,677, and 1,711 differentially expressed genes (DEGs), respectively. Of these, 3,005, 3,050, 1,608, 2,645, 1,792, and 549 genes were up-regulated, while 1,571, 6,923, 4,237, 8,640, 6,885, and 1,162 genes were down-regulated in response to infection. These results clearly demonstrate that SR infection induces significant changes in plant gene expression patterns (**Figure 5A**; **Supplementary Figure S5**).

GO enrichment analysis of biological processes, cellular components, and molecular functions indicated that enriched genes were involved in the regulation of DNA-templated transcription, nucleus, and ATP binding in response to SR infection (**Figure 5B**). KEGG enrichment analysis of DEGs revealed that the response of *A. albus* to SR infection at the transcriptional level was primarily associated with plant hormone signal transduction, plant-pathogen interactions, and ribosome-related resistance metabolites (**Figure 5C**). GSEA analysis identified 30 biological pathways and gene sets that were differentially activated at different time points during SR infection, with 13 showing a high level of enrichment and 17 showing a lower level of enrichment (**Figure 5D**).

### 3.7 Metabolome analysis of *A. albus* responsive to SR infection

**Figure 6:**
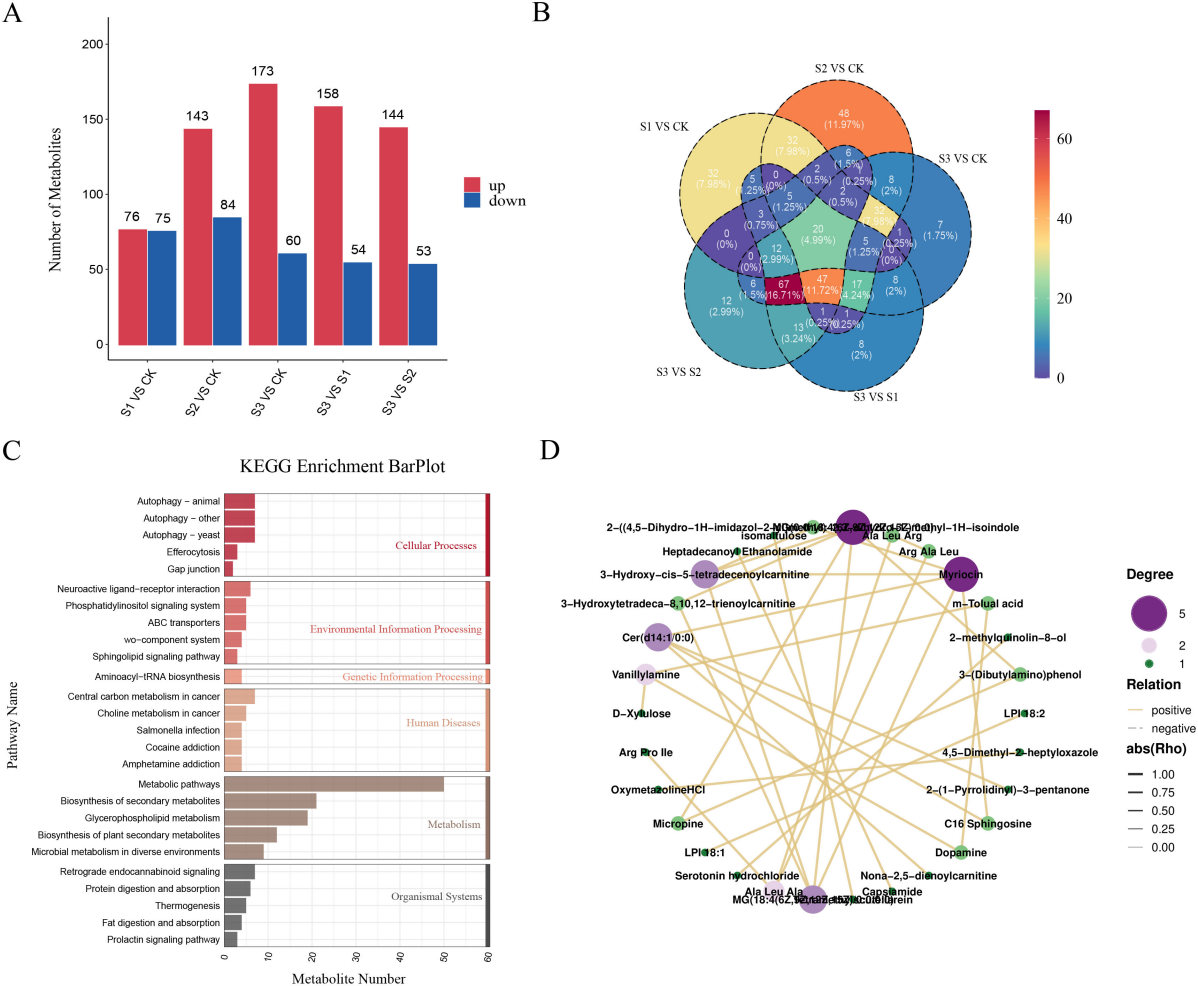
Metabolome analysis of *A*. *albus* responsive to SR infection during different stages (0h, 12h, 24h, 48h). **(A)** Differentially expressed metabolites (DEMs) of *A*. *albus* responsive to SR infection. This panel shows the metabolites that exhibit significant changes in their levels at various time points (0h, 12h, 24h, and 48h) following SR infection. **(B)** Venn diagram for DEMs from pairwise comparisons. This panel displays the overlap and unique DEMs identified through pairwise comparisons between different time points (e.g., 0h vs 12h, 12h vs 24h, 24h vs 48h). **(C)** KEGG enrichment analysis with DEMs of *A*. *albus* responsive to SR. This panel shows the KEGG pathway enrichment analysis for the differentially expressed metabolites (DEMs) identified in *A*. *albus* in response to SR infection. **(D)** Network graph for DEMs. This panel represents the interactions between the differentially expressed metabolites (DEMs) in *A*. *albus* during SR infection.

Metabolites in the leaves of *A. albus* were detected using LC-MS/MS non-targeted metabolomics at various stages of SR infection. Comparative analysis across five groups of differentially expressed metabolites (DEMs) (S1 vs. CK, S2 vs. CK, S3 vs. CK, S3 vs. S1, and S3 vs. S2) identified 151, 327, 233, 202, and 198 metabolites, respectively. Among these, 76, 143, 173, 158, and 144 metabolites were up-regulated, while 75, 84, 60, 64, and 53 metabolites were down-regulated in response to infection (**Figure 6A**). Venn diagram analysis further revealed 20 common DEMs across the five comparisons, suggesting their potential role in resistance to SR infection (**Figure 6B**). KEGG pathway analysis indicated that these DEMs were enriched in various pathways, including metabolic pathways, biosynthesis of secondary metabolites, and glycerophospholipid metabolism (**Figure 6C**). The network visually represents the interactions among differentially expressed metabolites (DEMs) in *A. albus* during infection, highlighting key DEMs and potential regulatory relationships that may play a significant role in response to SR infection. (**Figure 6D**).

### 3.8 Combined transcriptome and metabolome analysis

**Figure 7:**
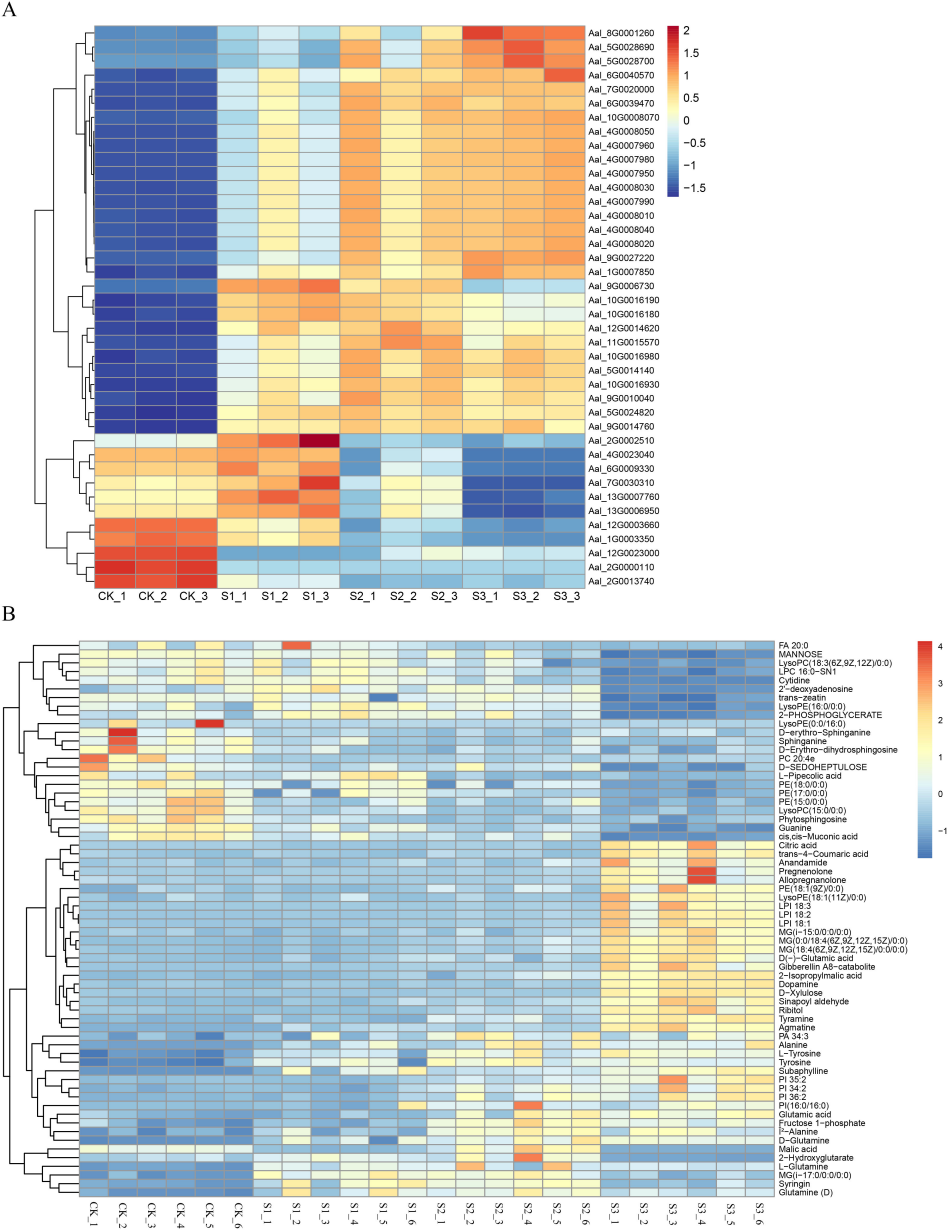
Heat maps and KEGG pathway enrichment diagrams of DEGs and DEMs. **(A)** Heat maps of DEGs in different infected stages. This panel presents the heat maps of differentially expressed genes (DEGs) at various stages of SR infection (e.g., 0h, 12h, 24h, 48h). **(B)** Heat maps of DEMs in different infected stages. This panel displays the heat maps of differentially expressed metabolites (DEMs) at different time points during SR infection (e.g., 0h, 12h, 24h, 48h).

**Figure 8:**
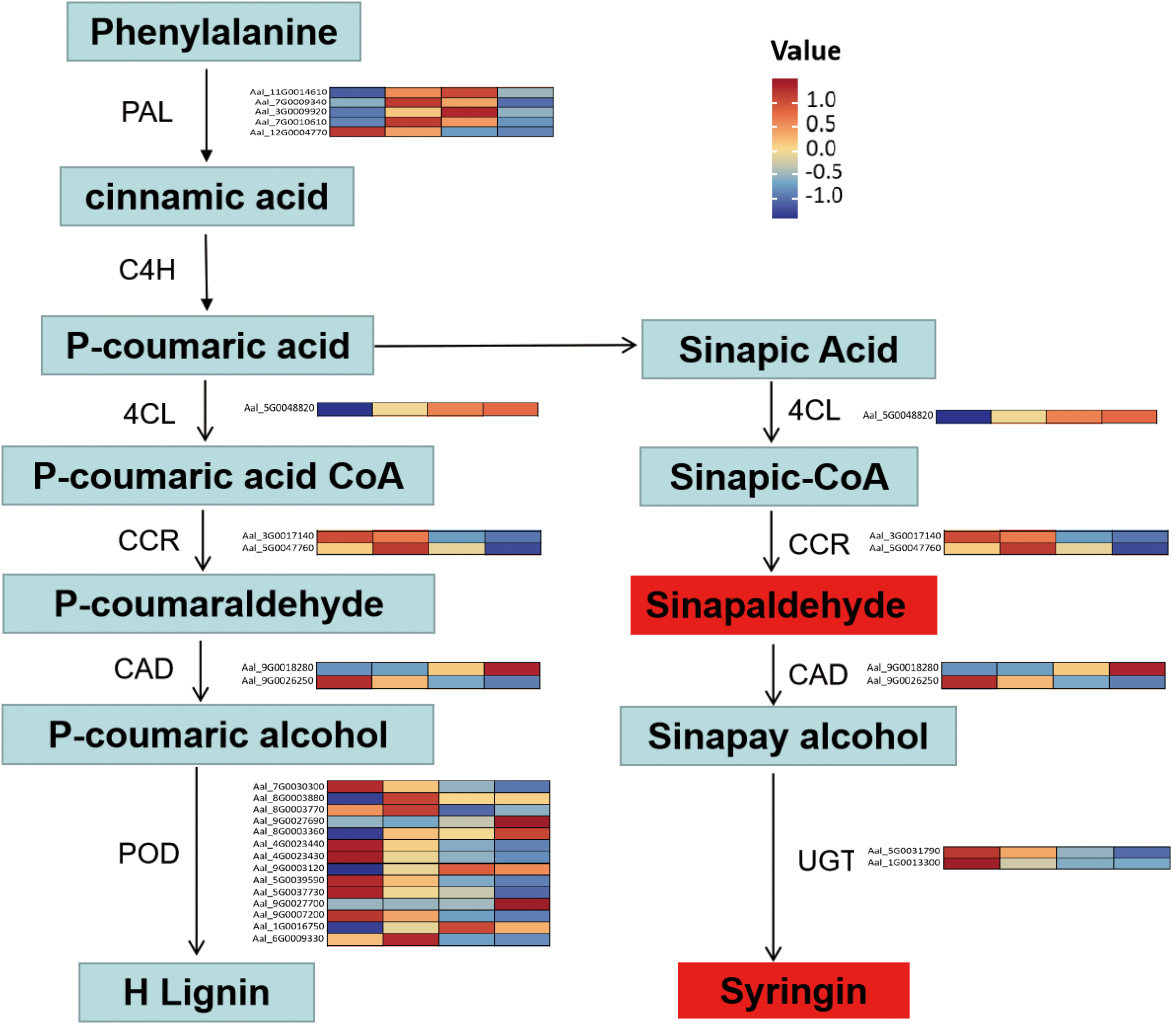
Expression profiles of genes and metabolites involved in the Phenylpropanoid biosynthesis pathway. The heat map shows the upregulation or downregulation of genes related to the synthesis of phenylpropanoid compounds.

According to the DEM analysis results of this experiment, combined with the transcriptome analysis results of the DEG, the results showed that 124 pathways were co-enriched (**Supplementary Figure S6**). The correlations between the top 41 DEGs and 62 DEMs were selected and represented as a heat map (**Figures 7A, B**). Coenrichment annotations of DEGs and DEMs were carried out, the results of which indicated involvement in Phenylpropanoid biosynthesis, Plant hormone signal transduction, Phenylalanine metabolism, Phenylalanine and tyrosine and tryptophan biosynthesis. Meanwhile, some key metabolites (trans-4-Coumaric acid, Tyrosine, Sinapoyl aldehyde, Syringin,trans-zeatin,Tyrosine,L-Tyrosine,and Fructose 1-phosphate) in related metabolic pathways were accumulated (**Figure 8**; **Supplementary Table S8**).

## 4 Discussion

In this study, we present the first chromosome-level genome assembly of *A. albus*, providing crucial molecular resources for understanding this economically important species. The assembled genome (5.9 Gb) is slightly larger than that of *A. konjac* (5.6 Gb), though it contains fewer protein-coding genes (35,155 versus 44,333) (Gao et al., 2022). This difference in gene content despite similar genome sizes suggests distinct evolutionary trajectories between these species (Wang et al., 2021). Comparative genomic analyses revealed that *A. albus* and *A. konjac* diverged approximately 1.3 million years ago, forming a monophyletic clade (Liu et al., 2019). This close phylogenetic relationship explains their successful interspecific hybridization in current breeding programs (Anil et al., 2023). Beside both *A. albus* and *A. konjac* have undergone a WGD event, significantly influencing their genome evolution (Li et al., 2023). Notably, we observed contrasting patterns in gene family dynamics between the species, with *A. konjac* showing unique expansion patterns and recent transposable element bursts contributing to its genome size (Hong et al., 2021). These findings provide insights into the molecular basis of species-specific traits and adaptation mechanisms (Fang and Wu, 2004).

Previous studies have demonstrated that KGM content varies significantly among different *Amorphophallus* species and is influenced by both genetic and environmental factors (Diao et al., 2014; Smith et al., 2018). The biosynthesis of KGM involves complex metabolic pathways, including the activation of mannose and glucose precursors, and is regulated by multiple enzymes (Karasov et al., 2017). A particularly interesting finding is the high KGM content in *A. albus* despite its lower biomass yield compared to *A. konjac*. This observation suggests a fundamental trade-off between biomass production and metabolite accumulation, which has important implications for breeding strategies (He et al., 2022). Similar trade-offs between primary metabolism and secondary metabolite production have been documented in various medicinal plants (Jian et al., 2021) and crop species (Vyska et al., 2016), reflecting the complex resource allocation patterns in plant metabolism. Understanding the genetic basis of this trade-off could be crucial for developing varieties that optimize both yield and KGM content, particularly given the increasing market demand for high-quality KGM products (He et al., 2016).

The effective control of plant diseases, particularly southern blight which is prevalent throughout *Amorphophallus* cultivation areas, can be achieved through enhanced plant resistance (Windram and Denby, 2015). Our study revealed that the plant defense response involves complex molecular mechanisms, including the MAPK cascade, plant hormone signaling, and secondary metabolite production. Notably, we observed that southern blight infection triggered large number of DEGs and DEMs in *A. albus*, with their numbers increasing over the infection period. This temporal pattern of transcriptional and metabolic reprogramming reflects the dynamic nature of plant-pathogen interactions (Gao et al., 2024). These findings align with previous studies on *A. konjac*, where Pcc infection induced the expression of genes involved in alkaloid metabolism pathways, particularly those related to shikimate-derived alkaloids and isoquinoline alkaloid biosynthesis (Kaur et al., 2022). The accumulation of these defense-related metabolites represents an important component of the plant’s chemical defense arsenal against pathogens (Xu et al., 2018).

Our integrated transcriptomic and metabolomic analyses revealed that multiple resistance-related pathways contribute to the defense mechanism of *A. albus* against southern blight pathogen. The coordinated regulation of these pathways demonstrates a complex and multi-layered defense response, reflecting the sophisticated evolution of plant immune systems (Han, 2019; Li et al., 2021). Similar to findings in other plant species, we observed the activation of phenylpropanoid biosynthesis pathway, which plays a crucial role in plant defense through the production of antimicrobial compounds and physical barriers (Kumar et al., 2023; Yang et al., 2023). The flavonoid biosynthesis pathway was also significantly upregulated, consistent with previous studies showing its importance in disease resistance in various crops (Ding et al., 2022). These defense responses involve both constitutive and induced mechanisms, with significant crosstalk between different signaling pathways (Varshney et al., 2021).

Additionally, future research should prioritize the functional characterization of candidate resistance genes and validate their roles in disease resilience. The integration of multiple omics approaches, including proteomics and metabolomics, would provide a more comprehensive understanding of the molecular mechanisms underlying disease resistance and KGM biosynthesis (Wang et al., 2022). Additionally, the application of advanced gene editing technologies could accelerate the development of superior cultivars with enhanced traits (Hillary and Ceasar, 2024). This study not only advances our understanding of *A. albus* biology but also provides a valuable framework for future genomic research and breeding efforts aimed at improving crop resilience and productivity in *Amorphophallus* species.

## 5 Conclusions

In this study, we present the first high-quality chromosome-level genome assembly of *A. albus*, comprising 5.94 Gb with a contig N50 of 5.61 Mb. The assembly achieved exceptional completeness, with 99.4% of sequences anchored to 13 chromosomes. Comprehensive annotation revealed 85.07% repetitive sequences and 39,938 protein-coding genes. Comparative genomic analysis identified a WGD event occurring approximately 8.418 million years ago, providing insights into the evolutionary history of *A. albus*. Integration of transcriptomic and metabolomic analyses unveiled significant enrichment of DEGs and DAMs involved in phenylpropane biosynthesis pathways. Notably, we identified several key genes metabolites conferring resistance to southern blight pathogen, which represent promising candidates for future molecular breeding programs. This high-quality reference genome not only provides a valuable resource for understanding the biological characteristics of *A. albus* but also establishes a robust foundation for molecular breeding efforts aimed at crop improvement.

## Data Availability

The genome assembly, raw sequencing reads of DNBseq, Pacbio, Hi-C data reported in this study have been deposited in the National Center for Biotechnology Information (NCBI) Bioproject database under the accession number PRJNA1208222. The transcriptome data has been uploaded to NCBI with the number GSM8721328- GSM8721339.
