# Supplementary material for: Genomic, transcriptomic and metabolomic analyses of Amorphophallus albus provides insights into the evolution and resistance to southern blight pathogen
Source: Front Plant Sci. 2025 Feb 7;15:1518058. doi: 10.3389/fpls.2024.1518058 (PMC11842328; doi:10.3389/fpls.2024.1518058)
Supplement: Supplementary file 1 [file SupplementaryFile1.docx]

Fig. S1 GO enrichment of contraction gene family


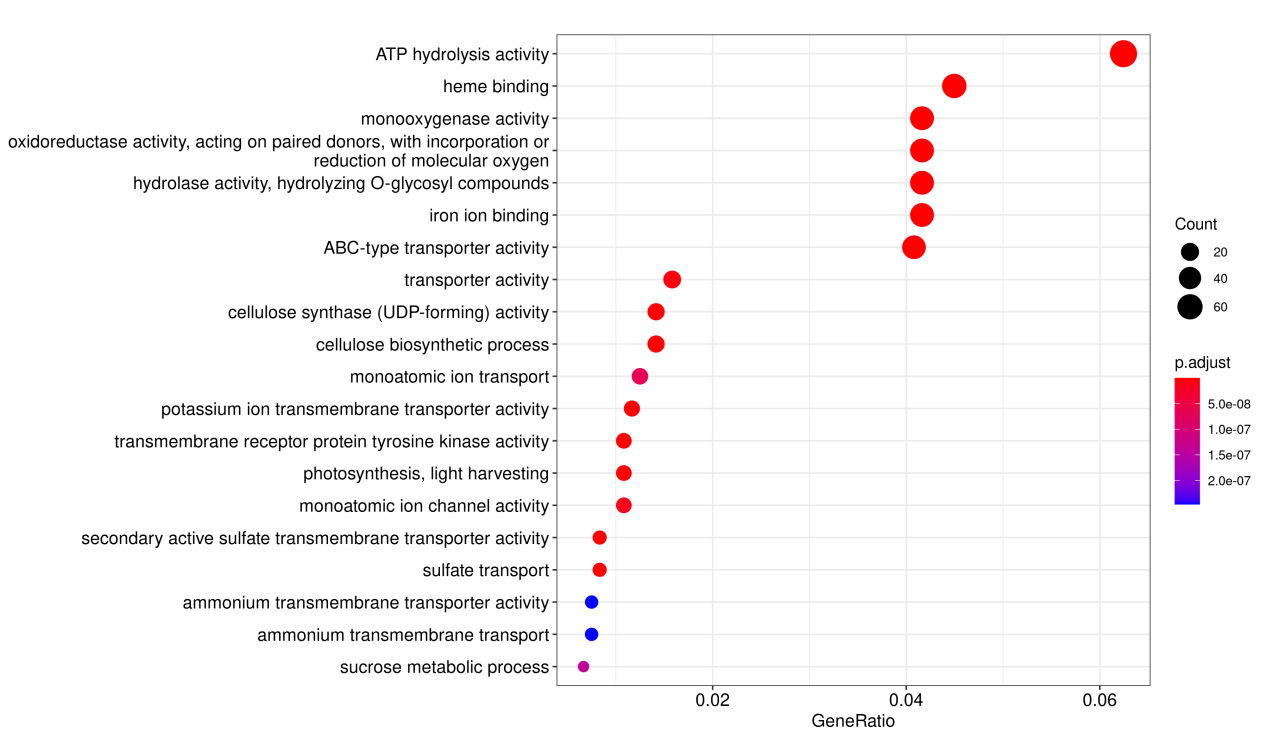


Fig.S2 GO enrichment of expansion gene family

**
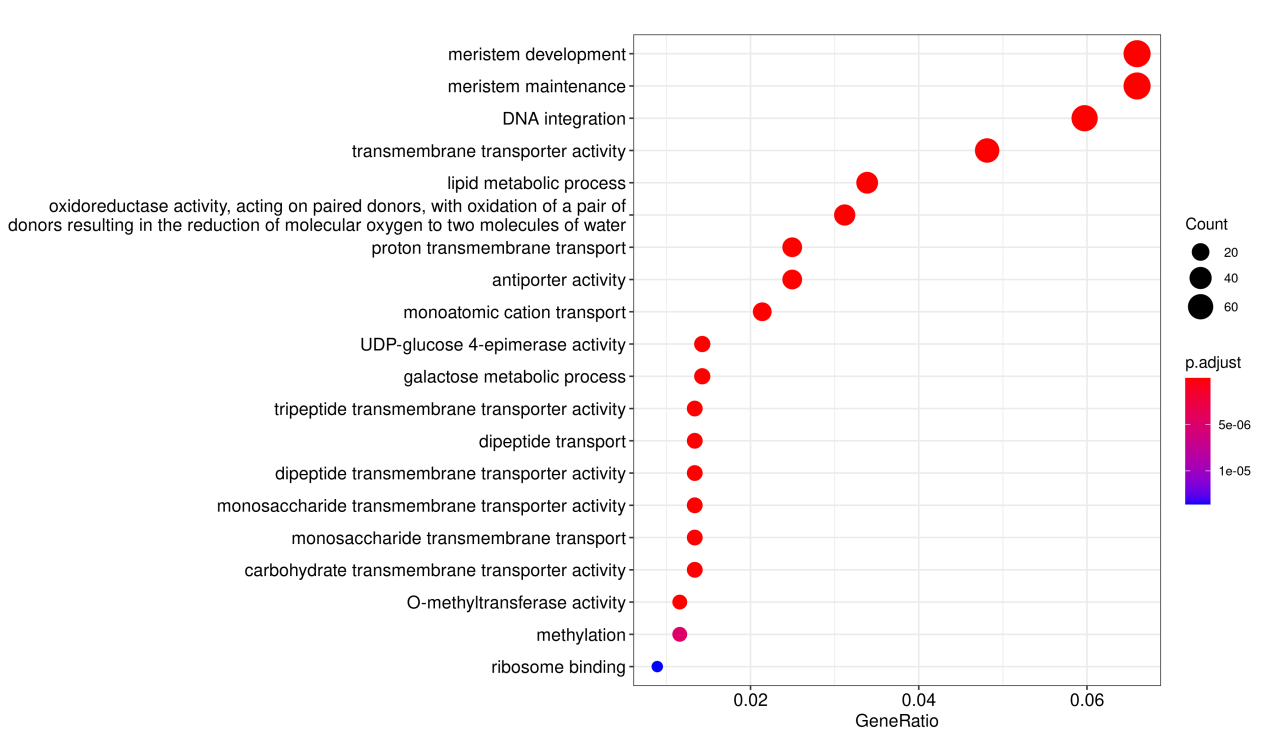
**

Fig.S3 KEGG enrichment of contraction gene family


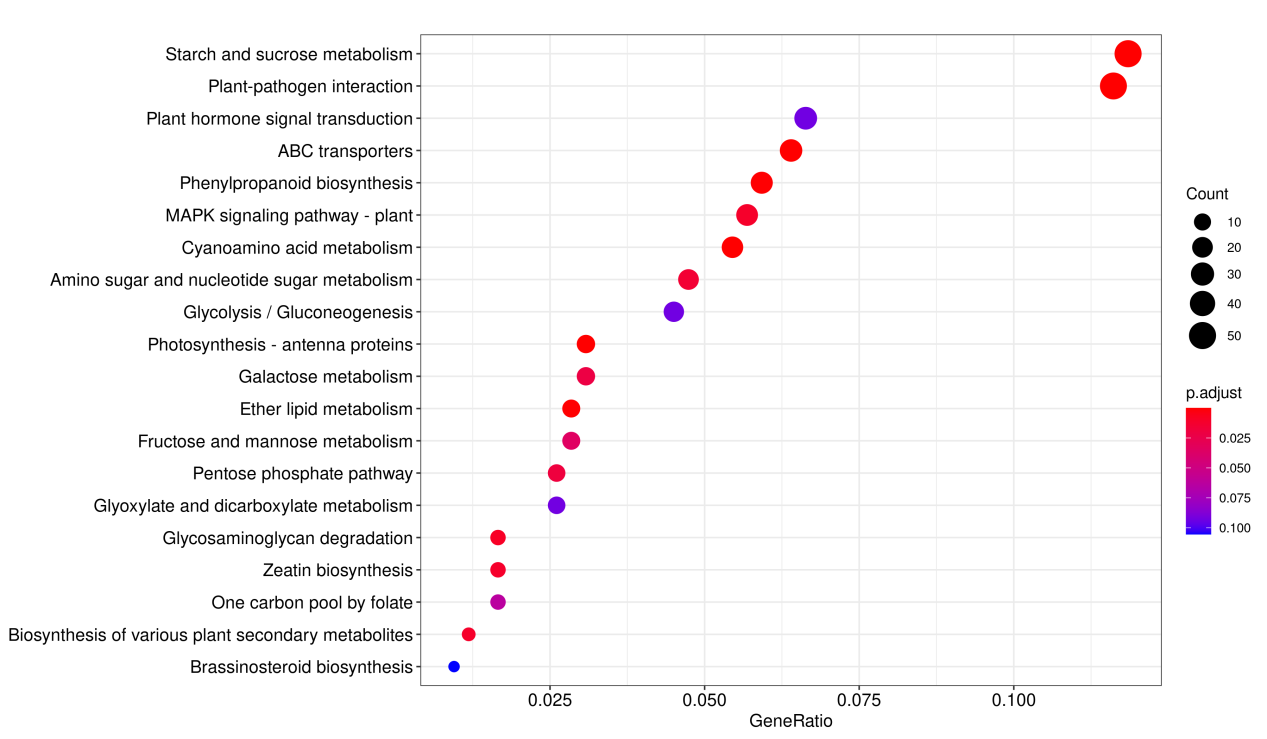


Fig.S4 KEGG enrichment of expansion gene family


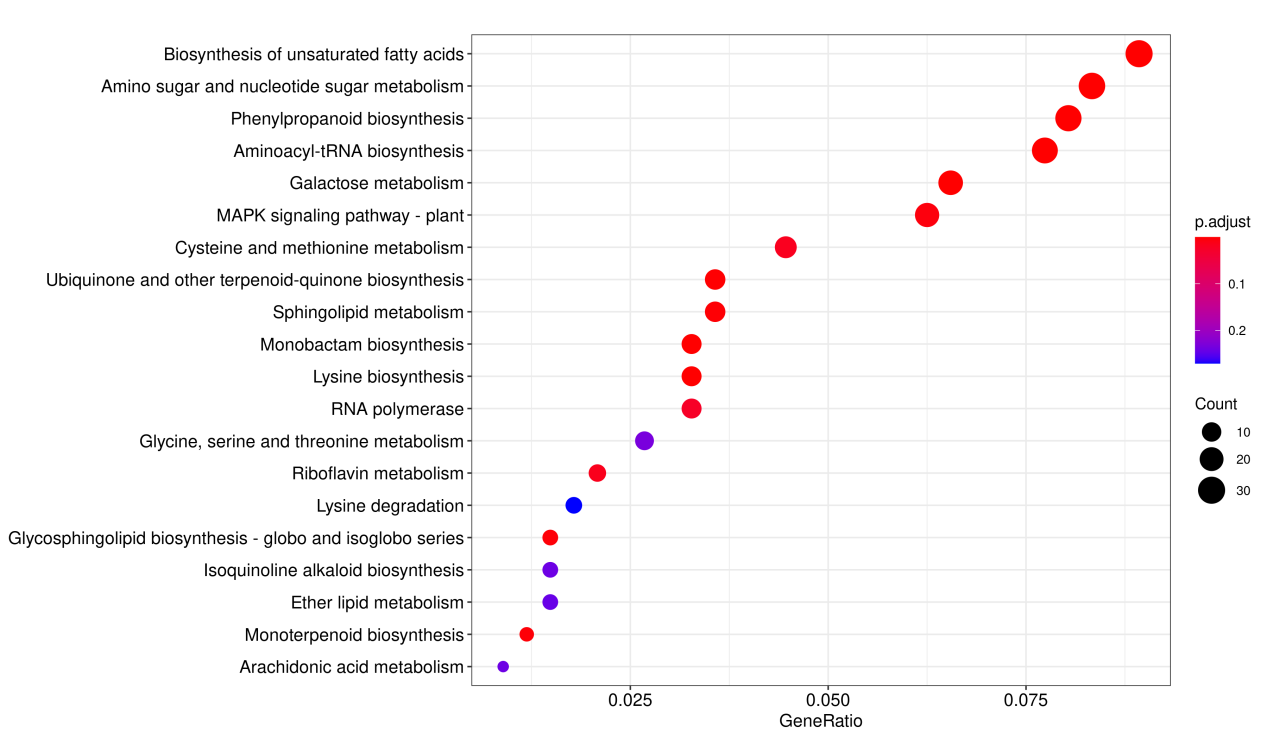


Fig.S5 Clustering Heatmap of Differentially Expressed Genes (DEGs) across different treatment Groups and Time Points


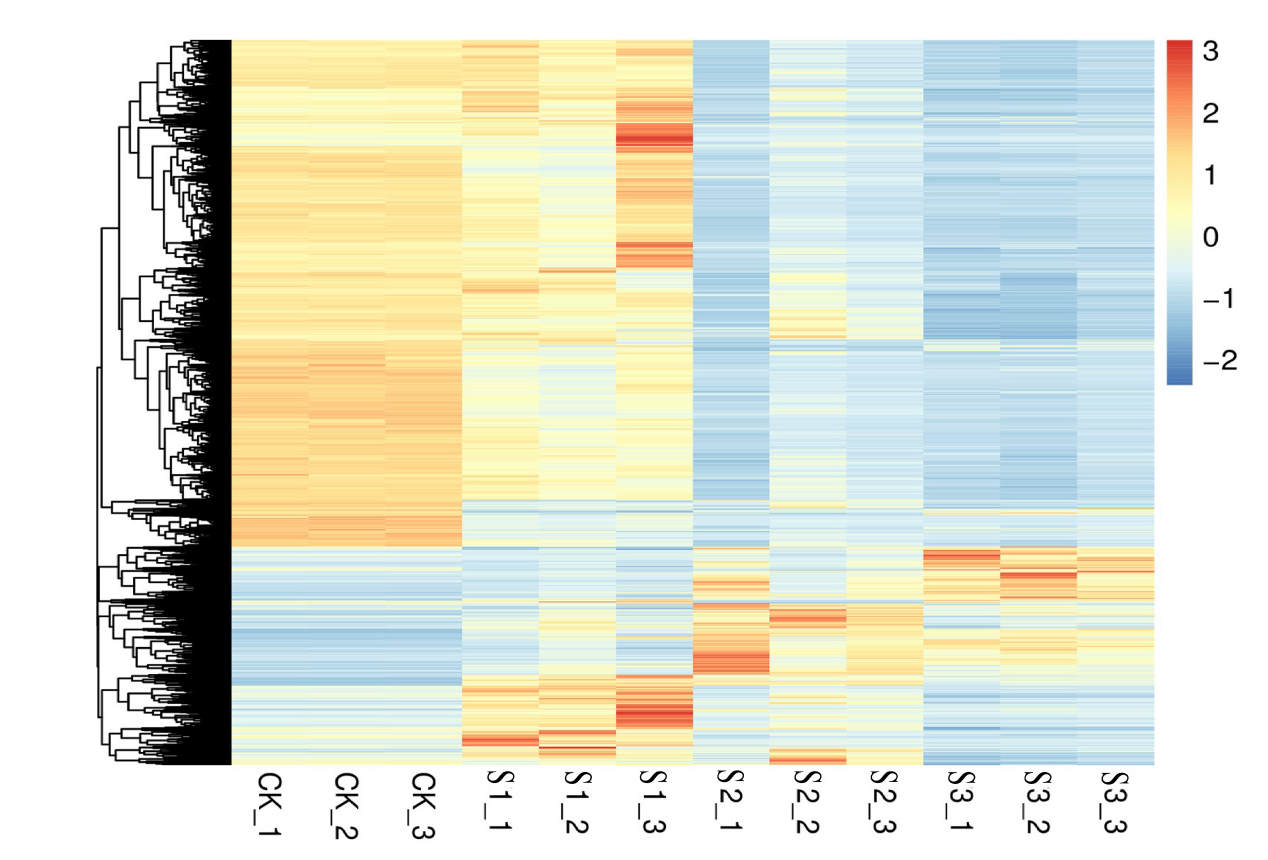


Fig.S6 Venn diagram of different pathwanys between genes and metabolites

**
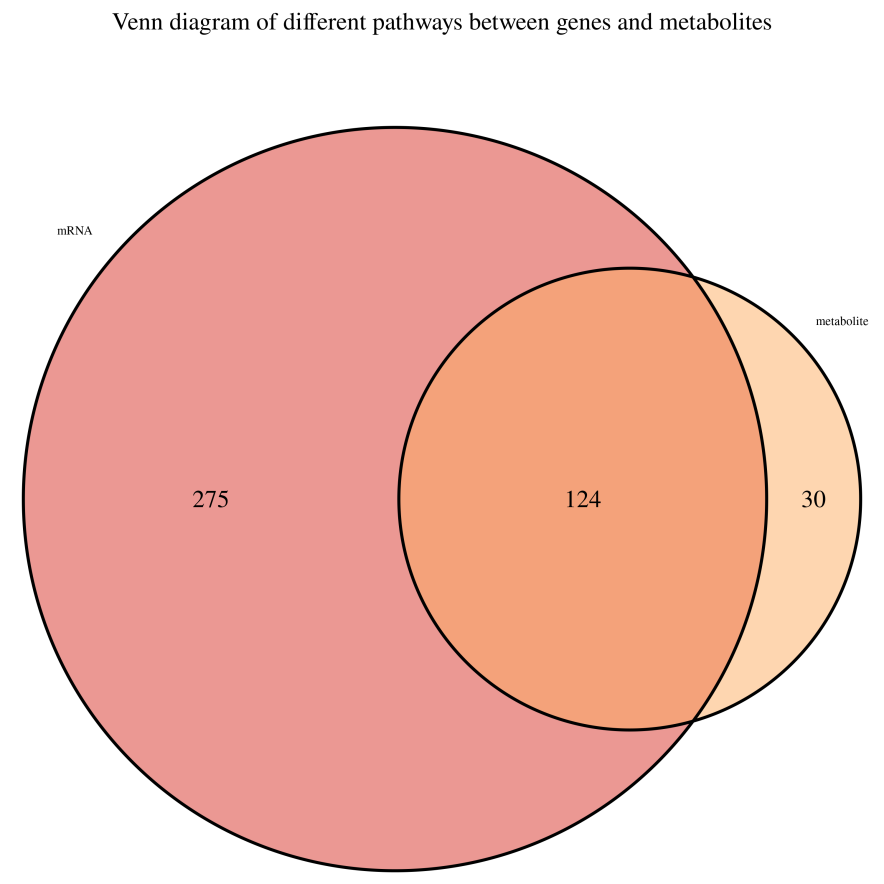
**
